# Qualitative Experiences of Multi-Sensory Engagement for Acutely Ill Persons With Dementia

**DOI:** 10.1093/geroni/igaa057.457

**Published:** 2020-12-16

**Authors:** Sheila Molony, Christine Waszynski

**Affiliations:** 1 Quinnipiac University, Hamden, Connecticut, United States; 2 Hartford Hospital, Hartford, Connecticut, United States

## Abstract

Delirium occurs in up to 25% to 50% hospitalized patients and the risk is increased for older adults with dementia. Multi-sensory stimulation has been effective in community and nursing home settings to reduce delirium in persons with dementia. This paper presents qualitative findings from field notes, observations and participant/family interviews. A sample of 25 individuals with dementia and were recruited from 2 medical units and randomized to intervention with two sessions in the multi-sensory “Hub” on 2 hospital days or receive usual care. One investigator recorded field notes prior to and during the intervention (or control). An RN in the Hub documented participant engagement. Audio-taped interviews with participants and family were recorded immediately after Hub visits. All data was transcribed and analyzed by two other team members using NVivo 12.0. Both coders reached consensus after independently coding the first 12 transcripts using open coding and descriptive content analysis. Pre-intervention data revealed common symptoms of delirium in both groups and post-intervention fatigue as a prominent theme. Participants in both groups were able to engage with the researcher and respond to interviews. Hub participants demonstrated high levels of engagement with some surprising positive responses and improvement from pre-intervention behavior. Qualitative findings from this pilot study highlight the abundance of delirium risk factors encountered by hospitalized older adults with dementia and demonstrate positive, engaging MMSE experiences, despite these risks. Further study is needed to identify longer-term impact.

